# MHC-Ig induces memory T cell formation *in vivo* and inhibits tumour growth

**DOI:** 10.1002/iid3.35

**Published:** 2014-11-11

**Authors:** Christian Schütz, Alessia Zoso, Shiwen Peng, Jonathon D Bennett, Jonathan P Schneck, Mathias Oelke

**Affiliations:** 1 Department of Pathology, The Johns Hopkins Institute of Cell EngineeringBaltimore, Maryland, 21205; 2 Diabetes Research Institute, University of MiamiMiami, Florida, 33136; 3 Department of Pathology, The Johns Hopkins Medical InstitutionsBaltimore, Maryland, 21205; 4 National Institutes of Health, National Institute on Aging, Biomedical Research CenterBaltimore, Maryland, 21224

**Keywords:** Adoptive T cell transfer, cancer, *in vivo* vaccination, memory T cells, MHC-Ig

## Abstract

Induction of a T cell mediated immune response is critical for the eradication of viral infections and tumours. Soluble peptide-loaded major histocompatibility complex-Ig (^pep−^MHC-Ig) have been shown to bind their cognate ligands, T cell receptor, with high affinity, and are successfully used to visualize antigen-specific T cells. Furthermore, immobilized ^pep−^MHC-Ig can activate and expand antigen-specific T cells in vitro and *in vivo*. In this study, we investigate the use of ^pep−^MHC-Ig as a potential strategy to modulate antigen specific T cell immune responses *in vivo*. ^SIY−^K^b^-Ig immunization, together with the pre-activation by an anti-CD40 monoclonal antibody, is able to stimulate a strong expansion of adoptively transferred 2C transgenic T cells and the formation of long term antigen-specific memory T cells. In addition, mechanistic studies show that the ^pep−^MHC-Ig molecules directly activate T cells *in vivo* without requiring uptake and reprocessing by antigen-presenting cells. Furthermore, B6 mice immunized with ^pep−^MHC-Ig molecules inhibit tumour growth in a B16-SIY melanoma prevention model. Thus, soluble ^pep−^MHC-Ig molecules represent a powerful tool for active immunotherapy.

## Introduction

The major goal of immunotherapy is to design new vaccines aimed at stimulating a protective T cell based immune response against viral infection and cancer. Generation of an effective cytotoxic T lymphocyte (CTL) immune response requires a minimum of two signals: one antigen-specific signal provided through MHC-TCR interaction and a second signal, which is provided by co-stimulatory molecules, such as CD28 on T cells and its ligand B7 family members on antigen-presenting cells (APCs). Absence of co-stimulatory signaling generally causes T cells to be in a non-responsive state such as with immature dendritic cells (DC) that express only low levels of co-stimulatory molecules. Therefore DC maturation is critical for successful T cell activation and is often accomplished through use of adjuvants that induce maturation. In a normal immune reaction activated CD4^+^ T cells up-regulate CD40L, inducing maturation in CD40-expressing APC and consequently resulting in up-regulation of Fcγ receptors and co-stimulatory molecules such as B7 family members. It has been shown both in vitro and *in vivo* that cross linking CD40 on APC using anti-CD40 antibodies mature DC, bypassing the requirement of CD4^+^ T cell help during the generation of CTL immune responses [1–4]. While, it has been shown that short term antigen stimulation and subsequent induction of CTL responses is feasible in the absence of CD4^+^ T cell help [5–10], CD4^+^ T cell help is required to generate functional memory T cells that are capable to faster respond to antigen re-challenge [11–14].

Development of soluble multivalent ^pep−^MHC complexes has enabled the direct visualization of antigen-specific T cells and has significantly increased the understanding of T cell mediated immune responses. In addition, it has been shown that soluble MHC class I dimer and tetramers can be used to modulate immune responses [15–17] and activate naïve CD8^+^ T cells in vitro even in the absence of co-stimulation or exogenous growth factor [18,19]. *In vivo*, however, administration of multiple doses of soluble ^pep−^MHC has lead to clonal exhaustion, energy or tolerance of the targeted T cells [20,21]. One of the soluble multivalent class I MHC-Ig dimer molecules that bind their cognate T cell receptor (TCR) with high affinity has been developed in our lab. These dimeric ^pep−^MHC-Ig complexes have been successfully used to visualize antigen-specific T cells [22–25]. While immunological function of classical peptide antigens is dependent on uptake and reprocessing, dimeric ^pep−^MHC-Ig can bind to Fcγ receptors on mature APC resulting in direct T cell stimulation facilitated by anti-CD40 mAb mediated up-regulated co-stimulatory molecules. Furthermore, peptide antigens have been demonstrated to induce T cell dysfunction and deletion at antigen rich vaccination sites when administered with incomplete Freud's adjuvant [26,27], favoring ^pep−^MHC-Ig as potential tool for immunotherapy.

In the current study, we investigated the potential of soluble ^pep−^MHC-Ig complexes as a tool for *in vivo* immunotherapy. We demonstrated that ^pep−^MHC-Ig dimer molecules can directly activate adoptively transferred antigen-specific T cells *in vivo*. In addition, pretreatment with anti-CD40 mAb greatly enhanced the *in vivo* potency of soluble ^pep−^MHC-Ig dimer. The stimulated CTL could lyse target cells even 30 days after ^pep−^MHC-Ig immunization and displayed a CD44^high^ and CD45^low^ memory phenotype. Moreover, we were able to induce a functional antigen-specific T cell response from the endogenous T cell repertoire in B6 mice immunized with ^pep−^MHC-Ig, which inhibited tumour growth in a B16-SIY melanoma model. Thus, soluble ^pep−^MHC-Ig dimer molecules hold great potential for the development of novel antigen-specific immunotherapeutic approaches.

## Results

### ^Pep−^MHC-Ig dimer molecules activate and expand adoptively transferred T cells in an antigen-specific manner

**Figure 1:**
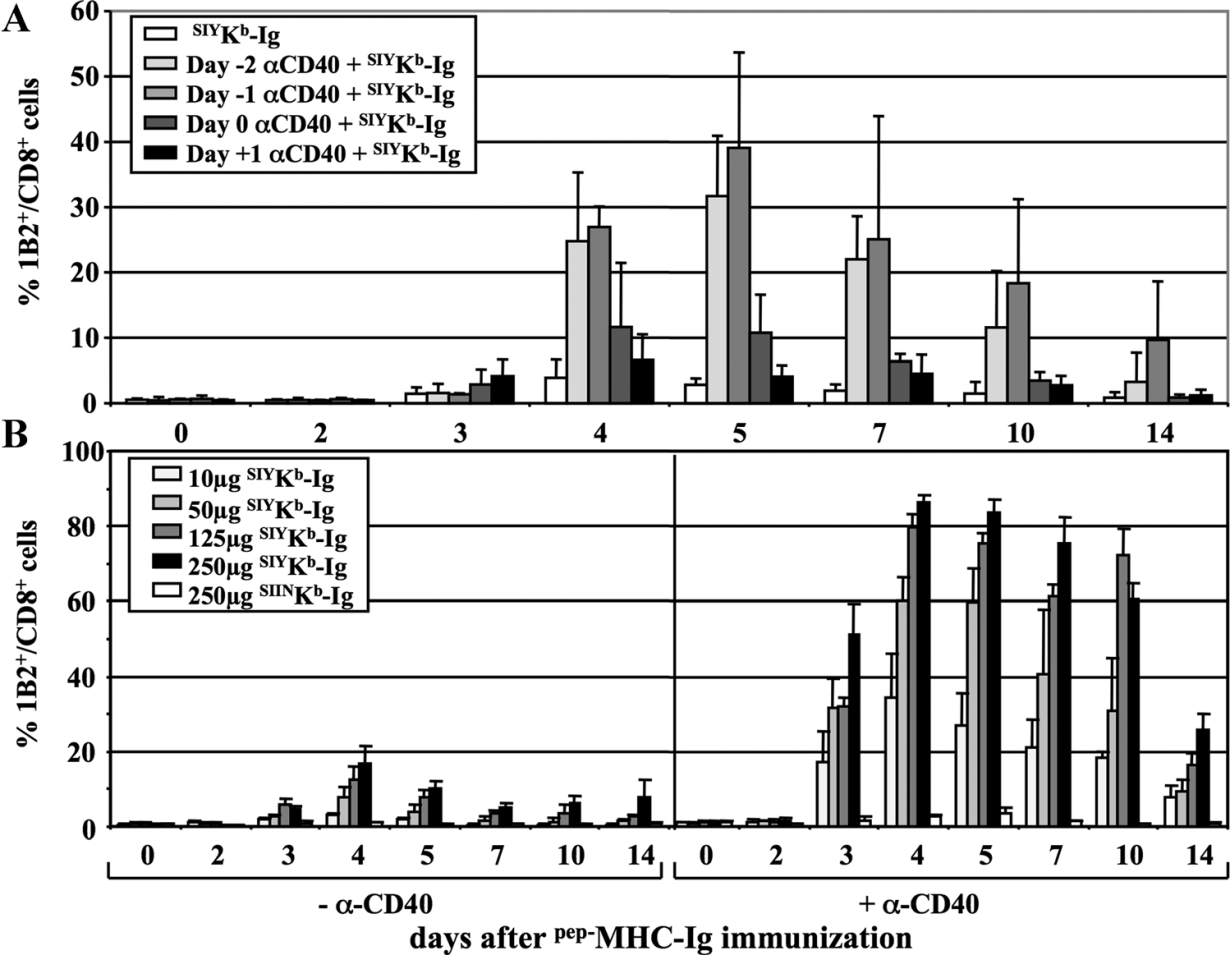
Anti-CD40 mAb pre-treatment; dose response and specificity of soluble ^SIY−^K^b^-Ig dimer immunization in adoptively transferred B6 mice. (A) Recipient mice were adoptively transferred with 3 × 10^6^ 2C T cells three days before immunization with 50 μg ^SIY−^K^b^-Ig dimer. On day −2, −1, 0, or +1 mice were i.p. injected with 10 μg/mouse anti-CD40 mAb. A group only immunized with dimer (^SIY−^K^b^-Ig) served as pre-treatment control. Peripheral blood was taken at the indicated time points and stained with anti-CD8 and anti-2C TCR clonotypic mAb (clone 1B2). Percentage (average ± SD) of 1B2^+^/CD8^+^ T cells/total CD8^+^ T cells is displayed. (B) Recipient mice adoptively transferred with 3 × 10^6^ 2C T cells, either pretreated with 10 µg/mouse anti-CD40 mAb (right half) or not pre-treated (left half), were immunized with s.c. injections of the indicated dose ^SIY−^K^b^-Ig dimer or ^SIIN−^K^b^-Ig dimer. Peripheral blood was taken at the indicated time points and stained with anti-CD8 and anti-2C TCR clonotypic mAb (clone 1B2). Percentage (average ± SE) of 1B2^+^/CD8^+^ T cells/total CD8^+^ T cells is displayed.

Mature APC express elevated levels of co-stimulatory molecules and Fcγ receptors. Therefore, we hypothesized that ^pep−^MHC-Ig presented on Fcγ receptors of mature APC would increase the immunization induced T cell response. To initiate host APC maturation and to determine the optimal time point for an effective anti-CD40 mAb pre-treatment we used an adoptive transfer model. On day −3 before SIY peptide-loaded MHC-Ig dimer (^SIY−^K^b^-Ig) immunization B6 mice were adoptively transferred with CD8^+^ T cells isolated from splenocytes of syngeneic 2C TCR transgenic mice recognizing the synthetic peptide SIY presented on K^b^ ligand. Co-staining of PBMC with anti-CD8 and clonotypic 1B2 mAb specific for the 2C TCR controlled for efficient transfer. On day −2, −1, 0, or +1 animals were i.p. injected with 10 μg/mouse anti-CD40 mAb. Pre-treatment was most efficient when given before rather than after the ^SIY−^K^b^-Ig immunization with its peak at day −1 resulting in an expansion of up to 40% of 1B2^+^/CD8^+^ 2C T cells at day 5 after immunization. ^SIY−^K^b^-Ig immunization before or without anti-CD40 mAb pre-treatment showed only limited expansion of adoptively transferred T cells (Fig. 1A). Thus these findings demonstrate that maturation of APC was only effective when animals were treated with anti-CD40 mAb before ^pep−^MHC-Ig immunization.

To investigate the use of ^pep−^MHC-Ig to induce antigen-specific T cell immune responses *in vivo* in more detail, mice were adoptively transferred with SIY specific 2C T cells and 3 days later immunized s.c. with increasing amounts of ^SIY−^K^b^-Ig (Fig. 1B). Immunization with ^SIY−^K^b^-Ig induced a dose dependent expansion of adoptively transferred 2C T cells, with a maximum of 18% on day 4 after immunization with the highest dose of 250 μg. In contrast no expansion of 2C T cell was observed after immunization with 250 μg non-cognate, ^SIIN−^K^b^-Ig dimer (Fig. 1B, left hand).

To provide additional T cell help through increased expression of co-stimulatory molecules on mature dendritic cells, mice were treated with anti-CD40 mAb one day prior to immunization. As shown in Figure 1B anti-CD40 mAb pretreated mice exhibited significantly higher expansion levels of the adoptively transferred cells (Fig. 1B, right hand). For example, on day 4 we observed up to 87% 2C T cells in anti-CD40 mAb pre-treated animals. Furthermore, on day 4 in pre-treated animals, immunization with only 10 μg ^SIY−^K^b^-Ig dimer exceeded the maximum response seen in animals without anti-CD40 pre-treatment by about 100% (36% and 18%, respectively). No specific expansion was observed in mice pre-treated with anti-CD40 mAb and immunized with non-cognate control ^SIIN−^K^b^-Ig dimer, confirming that the induced T cell response was antigen-specific. Based on these findings we performed all *in vivo* experiments using anti-CD40 mAb pre-treatment.

### ^Pep−^MHC-Ig expanded CD8^+^ 2C T cells are activated and functional

**Figure 2:**
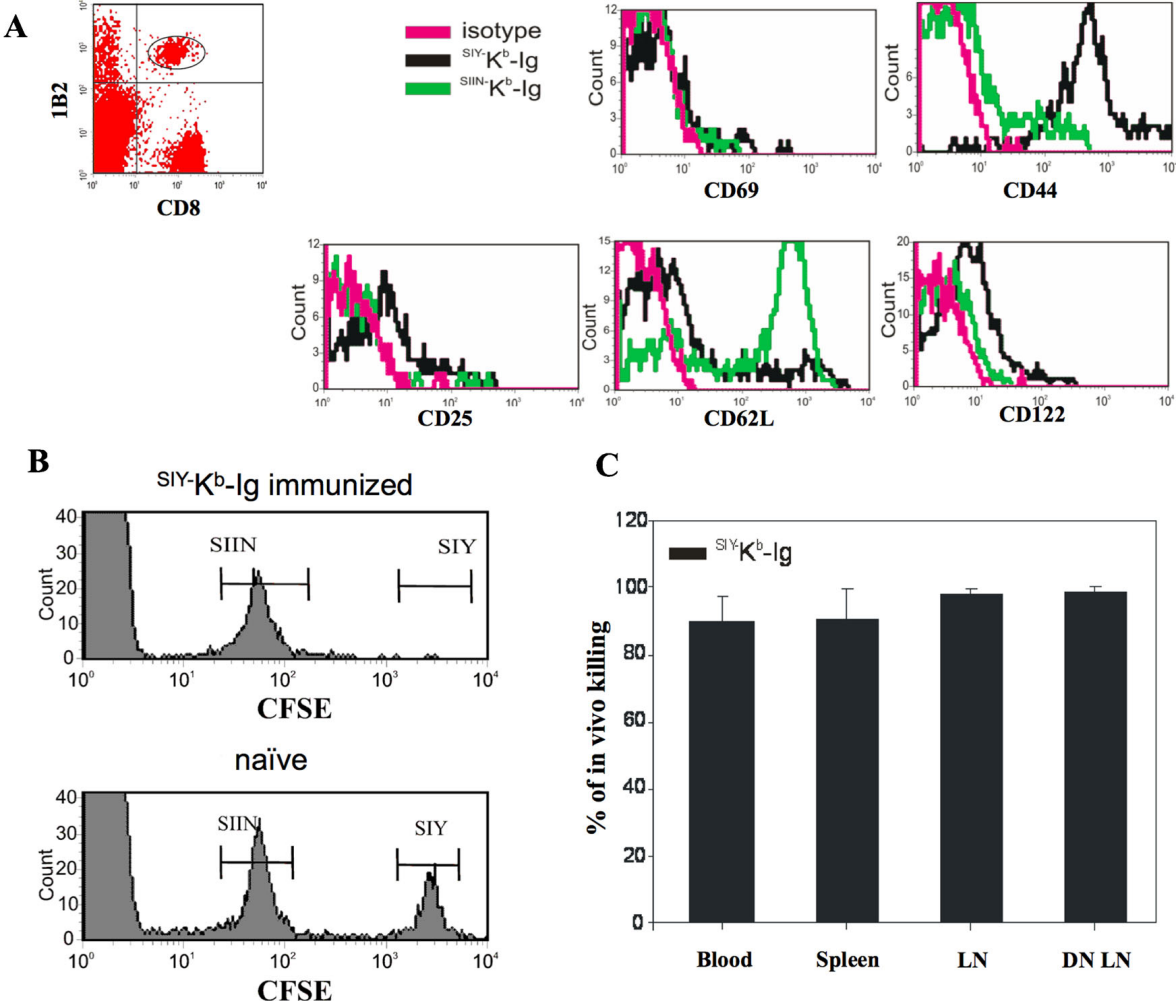
Phenotypic and functional characterization of adoptively transferred 2C T cells after ^pep−^MHC-Ig immunization. (A) 3 × 10^6^ purified 2C T cells were adoptively transferred into C57BL/6 mice. On day 2 after transfer, the recipient mice were treated with 10 µg/mouse anti-CD40 mAb and on day 3 immunized with ^SIY−^ K^b^-Ig or ^SIIN−^K^b^-Ig s.c. Peripheral blood was taken from the recipient mice at day 5 after immunization and stained with anti-CD8 and anti-2C TCR clonotypic mAb (clone 1B2) in combination with either anti-CD25, anti-CD44, anti-CD62L, anti-CD69, anti-CD122, or with the appropriate antibody isotype control. For analysis whole PBMC were first gated on 1B2^+^/CD8^+^ and further analyzed for activation marker expression. (B) *In vivo* CTL killing assay for adoptively transferred 2C T cells. Five days after immunization with ^SIY−^K^b^-Ig (upper panel) or naïve animals (lower panel) were injected with 1 × 10^7^ syngeneic splenocytes consisting of both an SIY peptide-pulsed, 2.5 µM CFSE labeled population and an SIIN peptide-pulsed, 0.25 µM CFSE labeled population (1:1 ratio). Eighteen hours later, peripheral blood was taken and analyzed for CFSE labeled cells by flow cytometer. One representative mouse each was displayed. (C) Cumulative *in vivo* killing data after ^SIY−^K^b^-Ig immunization separated by organ. Data have been calculated as % of *in vivo* killing = 100 − ([(% specific peptide pulsed cells in immunized B6/% unspecific peptide pulsed cells in immunized B6)/(% peptide pulsed in naïve B6/% unspecific peptide pulsed cells in naïve B6)] × 100) and presented as (average ± SD).

To characterize the ^pep−^MHC-Ig induced antigen-specific T cell responses we analyzed the phenotype of the stimulated T cells. Adoptively transferred cells were co-stained for the expression of CD25, CD44, CD62L, CD69, and CD122. 2C T cells from ^SIY−^K^b^-Ig immunized mice displayed an activated phenotype (CD25^+^, CD44^high^, CD62L^low^ and early activation marker CD69^−^, and CD122^−^) whereas the 2C T cell population from ^SIIN−^K^b^-Ig immunized mice had still a typical naïve phenotype (CD25^−^, CD44^low^, CD62L^high^, and early activation marker CD69^−^ and CD122^low^; Fig. 2A).

Cytotoxic function of the *in vivo* stimulated 2C T cells was analyzed by an *in vivo* CTL killing assay. B6 syngeneic splenocytes were pulsed with either cognate SIY or non-cognate SIIN peptide and labeled with either a high or low concentration of CFSE, respectively (^SIY−^CFSE^high^, ^SIIN−^CFSE^low^), mixed at a ratio of 1:1 and subsequently injected into B6 mice 5 days after immunization. The relative frequencies of the two CFSE labeled cell populations were determined by flow cytometry in ^SIY−^K^b^-Ig immunized and in naïve animals to calculate specific killing in blood, spleen, draining lymph node (DN LN), and non-draining lymph node (LN) (Fig. 2B and C). Approximately 90% specific killing was detected in all organs of the immunized mice. In contrast, no cytotoxic activity was detected against the non-cognate ^SIIN−^CFSE^low^ target population and only minimal *in vivo* killing was observed in control animals (Fig. 2B and C).

Together, these data demonstrate that ^SIY−^K^b^-Ig dimer immunization not only activates adoptively transferred 2C T cells *in vivo* in an antigen specific manner but also generates a functional immune response sufficient to kill cognate target cells.

### Activation of antigen-specific CD8^+^ T cells by dimeric ^pep−^MHC-Ig complex is not dependent on cross presentation

There are several potential mechanisms to explain how soluble dimeric ^pep−^MHC-Ig complexes activate antigen-specific T cells: direct interaction with TCR on the surface of CD8^+^ T cells, internalization, processing and presentation of dimer molecules by endogenous APC (cross-presentation), or simply through peptide shedding. Peptide shedding and exchange is unlikely as the amounts of peptide loaded onto dimer molecules are very small and experiments utilizing these minute amounts of peptides have shown no antigen-specific T cell responses (data not shown). To distinguish between the other two major mechanisms, we took advantage of the fact that the 2C TCR recognizes not only the synthetic peptide SIY presented on K^b^ ligand but also the allogeneic QL9 peptide presented in the context of L^d^ MHC-I molecules, which cannot be cross presented as B6 mice do not express L^d^ MHC-I molecules. Therefore, 2C T cells were adoptively transferred in B6 mice pre-treated with anti-CD40 mAb and immunized with either syngeneic ^SIY−^K^b^-Ig or allogeneic ^QL9−^L^d^-Ig dimer. As negative controls B6 mice were immunized with either ^SIIN−^K^b^-Ig or ^mCMV−^L^d^-Ig dimer, which did not induce any 1B2^+^/CD8^+^ T cell expansion (data not shown).

**Figure 3:**
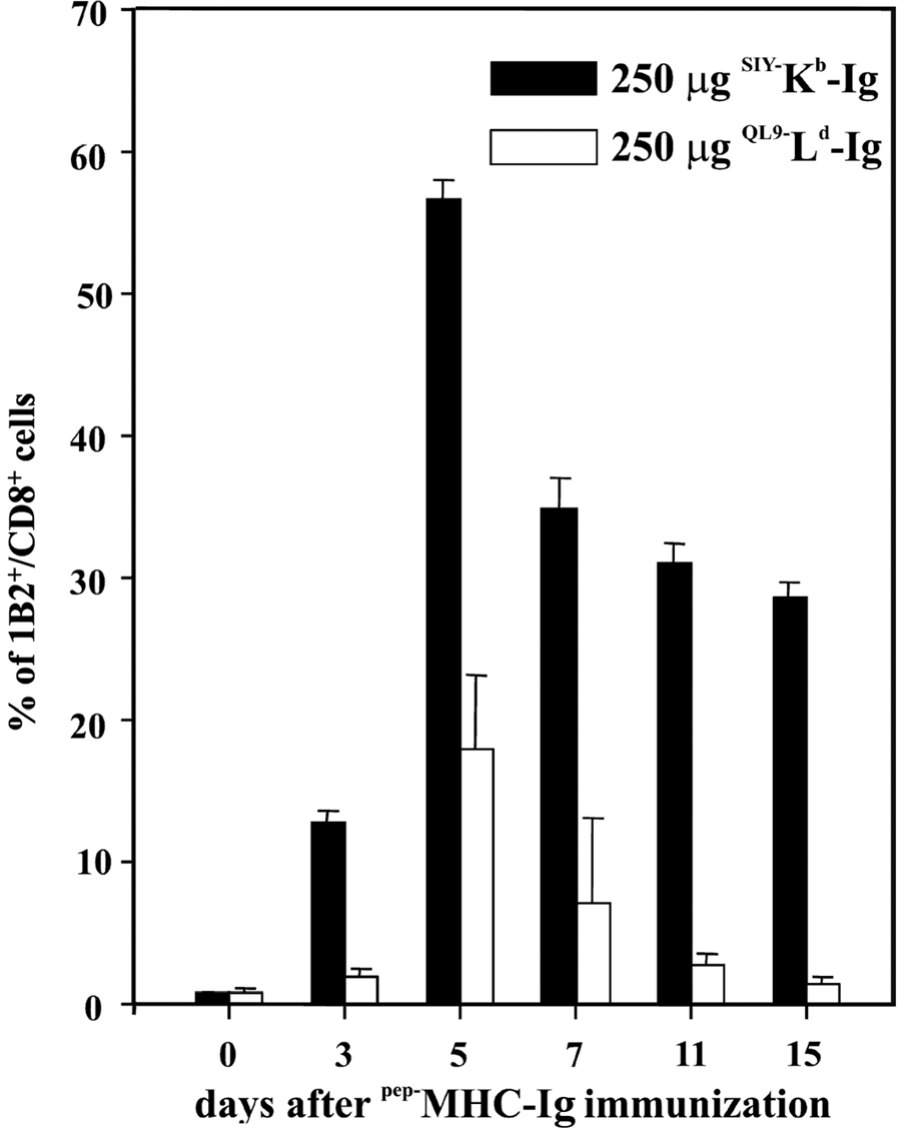
^pep−^MHC-Ig can directly interact with T cells *in vivo* to initiate antigen-specific T cell expansion. C57BL/6 mice were adoptively transferred with 3 × 10^6^ purified 2C T cells i.v. and s.c. immunized with the indicated dose of either ^SIY−^K^b^-Ig (filled bars) or ^QL9−^L^d^-Ig (open bars). Peripheral blood was taken from recipient mice at the indicated time points and stained with anti-CD8 and anti-2C TCR clonotypic mAb (clone 1B2). Percentage (average ± SE) of 1B2^+^/CD8^+^ T cells/total CD8^+^ T cells is displayed.

If cross-presentation were the only mechanism for induction of dimer T cell immune response, mice immunized with allogeneic ^pep−^MHC dimer should not show any 2C T cell expansion. As shown in Figure 3 both immunizations resulted in expansion of adoptively transferred 2C T cells, which peaked on day 5. ^QL9−^L^d^-Ig dimer immunization induced a maximum of 17% 2C T cells and ^SIY−^K^b^-Ig reached 55%, demonstrating that both the syngeneic and the allogeneic ^pep−^MHC dimer molecule, whose peptide cannot be cross-presented, induced robust proliferation of adoptively transferred 2C T cells. Thus we conclude that dimer immunization induced antigen-specific T cell stimulation through direct interaction with its cognate TCR after binding to Fcγ receptors on mature endogenous APC.

### ^SIY^^−^K^b^-Ig immunization induces a memory T cell response

**Figure 4:**
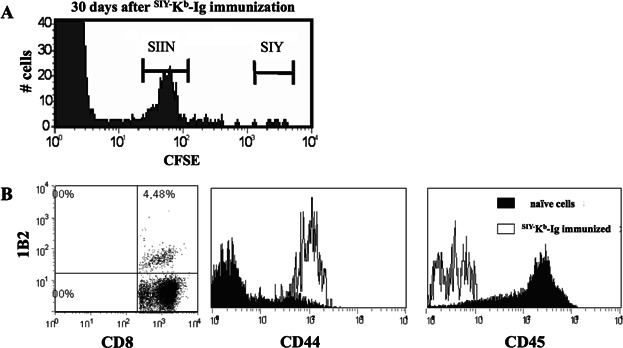
Induction of a memory immune response by ^SIY−^K^b^-Ig immunization. (A) *In vivo* CTL lysis assay 30 days after adoptive 2C T cell transfer into anti-CD40 mAb pretreated C57BL/6 mice. 1 × 10^7^ syngeneic splenocytes consisting of an SIY peptide-pulsed, 2.5 µM CFSE labeled population and an SIIN peptide-pulsed, 0.25 µM CFSE labeled population (1:1 ratio) were injected into recipient mice. Eighteen hours later, peripheral blood was taken from the recipient mice and CFSE labeled cells were analyzed by flow cytometer. (B) Two days following the 2nd ^SIY−^K^b^-Ig dimer immunization, peripheral blood was stained with anti-CD8, anti-2C TCR clonotypic mAb (clone 1B2, left plot), anti-CD44 (middle histogram) and anti-CD45 (right histogram). Naïve 1B2^+^/CD8^+^ cells isolated from C57BL/6 splenocytes served as a control.

Interestingly, even after 30 days from the initial dimer immunization we were still able to detect 1% 1B2^+^/CD8^+^ 2C T cells in the blood of ^SIY−^K^b^-Ig dimer immunized mice while control mice injected with ^SIIN−^K^b^-Ig complexes showed only background levels (data not shown). Therefore, we further characterize the immune response induced by a single dimer immunization with particular attention to a possible memory response. The residual population was functional and showed significant cytotoxicity against ^SIY−^CFSE^high^ labeled targets in mice immunized with ^SIY−^K^b^-Ig dimer (Fig. 4A).

We re-challenged the immunized mice with a second ^pep−^MHC dimer immunization 30 days after the initial immunization with an additional 250 µg. As expected for a memory T cell response, we observed that the secondary response was much faster than the primary response. In fact, the peak expansion for 1B2^+^/CD8^+^ T cells was reached within 2 days after the boost while the primary response required 5 (data not shown). Surface staining of the 1B2^+^/CD8^+^ T cells from these mice confirmed their CD44^high^ and CD45^low^ memory phenotype (Fig. 4B). Thus dimer immunization leads to the generation of a functional antigen-specific memory T cell response *in vivo* that is sufficient to induce antigen-specific killing even 30 days post immunization.

### ^SIY^^−^K^b^-Ig immunization of naïve mice induces a functional immune response

**Figure 5:**
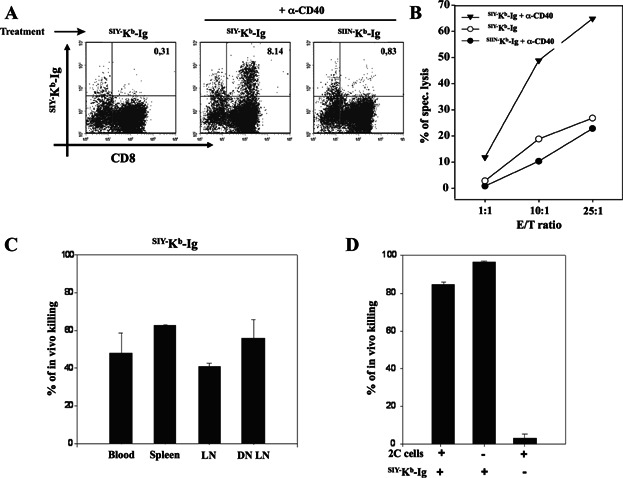
Generation of a functional antigen-specific endogenous T cell response in ^SIY−^K^b^-Ig immunized mice. (A) FACS analysis of splenocytes from C57BL/6J mice pre-treated with or without anti-CD40 mAb and immunized with 250 µg of either ^SIY−^K^b^-Ig or ^SIIN−^K^b^-Ig. Briefly, splenocytes from the immunized mice were harvested and in vitro stimulated for 5 days with SIY peptide pulsed T2-K^b^ cells. The cells were then stained with ^SIY−^K^b^-Ig, followed by anti-IgG1 and anti-CD8. (B) Splenocytes from immunized mice (as indicated in the legend) were in vitro re-stimulated with SIY peptide and tested in a ^51^Cr release assay with SIY peptide pulsed T2-K^b^ target cells. (C) *In vivo* killing assay. Five days after immunization with 250 µg ^SIY−^K^b^-Ig all mice were injected with 1 × 10^7^ cells consisting of a 1:1 mixture of SIY and SIIN peptide pulsed syngeneic splenocytes that were labeled with high (SIY) or low (SIIN) amounts of CFSE. Eighteen hours later peripheral blood, spleen, lymphnodes and draining lymphnodes were harvested from the mice and analyzed for CFSE labeled cells by flow cytometer. Data (average ± SD) shown were calculated as described in Material and Methods. (D) Analysis of splenocytes after an *in vivo* killing assay from C57BL/6J mice pre-treated with anti-CD40 mAb and immunized with 1mg ^SIY−^K^b^-Ig, with or without adoptively transferred 2C T cells (left and middle bar respectively). Non-immunized C57BL/6J mice adoptively transferred with 2C T cells (right bar) served as a negative control. Data shown as (average ± SD).

Having demonstrated the generation of a robust adoptively transferred 2C T cell response, we hypothesized that ^pep−^MHC-Ig dimer immunization of naïve B6 mice will result in a functional endogenous antigen-specific immune response. Naïve B6 mice were immunized with either ^SIY−^K^b^-Ig or ^SIIN−^K^b^-Ig with or without anti-CD40 mAb. To detect the endogenous response, we harvested splenocytes 30 days after immunization and than stimulated them ex vivo with SIY peptide pulsed T2-K^b^ cells (Fig. 5A). A significant expansion of SIY-specific CD8^+^ T cells were seen in ^SIY−^K^b^-Ig immunized mice pre-treated with anti-CD40 mAb (8.14%, Fig. 5A, center), while no sign of SIY-specific CD8^+^ T cell expansion was detected in splenocytes derived from ^SIIN−^K^b^-Ig immunized mice (0.83%, Fig. 5A, right hand side) or mice not pre-treated with anti-CD40 mAb (0.31%, Fig. 5A, left).

In vitro killing demonstrated that only CD8^+^ T cells derived from ^SIY−^K^b^-Ig immunized mice in combination with anti-CD40 mAb pre-treatment were able to kill SIY peptide pulsed T2-K^b^ target cells, after in vitro stimulation (Fig. 5B, filled triangles), while splenocytes from ^SIY−^K^b^-Ig alone, ^SIIN−^K^b^-Ig alone, or ^SIIN−^K^b^-Ig in combination with anti-CD40 mAb immunized mice showed only background levels of specific lysis (Fig. 5B, open circles and closed circles).

An *in vivo* killing assay was used to determine the functional effect of ^SIY−^K^b^-Ig immunization. We pre-treated mice with anti-CD40 mAb and immunized with 250 µg of ^SIY−^K^b^-Ig dimer; on day 5 after immunization we injected a mixture of ^SIIN−^CFSE^low^ and ^SIY−^CFSE^high^ labeled syngeneic splenocytes and evaluated antigen-specific killing in blood, spleen, LN and DN LN 24 h later. Around 50% killing was detected in all organs investigated (Fig. 5C). Moreover, the cytotoxic T cell response could be further augmented by increasing the amount of ^SIY−^K^b^-Ig dimer used for immunization. Thus, as shown in Figure 5D, immunization with 1 mg instead of 250 µg of ^SIY−^K^b^-Ig dimer resulted in an increased *in vivo* cytotoxicity from 50% (see Fig. 5C) to 90% (Fig. 5D, middle bar), which is comparable to *in vivo* killing in mice that were adoptively transferred with transgenic 2C T cells prior to immunization (Fig. 5D, left bar). In contrast, no cytotoxic activity was detected in B6 mice that were adoptively transferred with 2C T cells but received no dimer immunization (Fig. 5D, right bar). In summary, these data indicate that dimer immunization has the ability to induce a functional antigen-specific T cell response from the endogenous T cell repertoire.

### ^SIY^^−^K^b^-Ig immunization inhibits tumour growth *in vivo*

**Figure 6:**
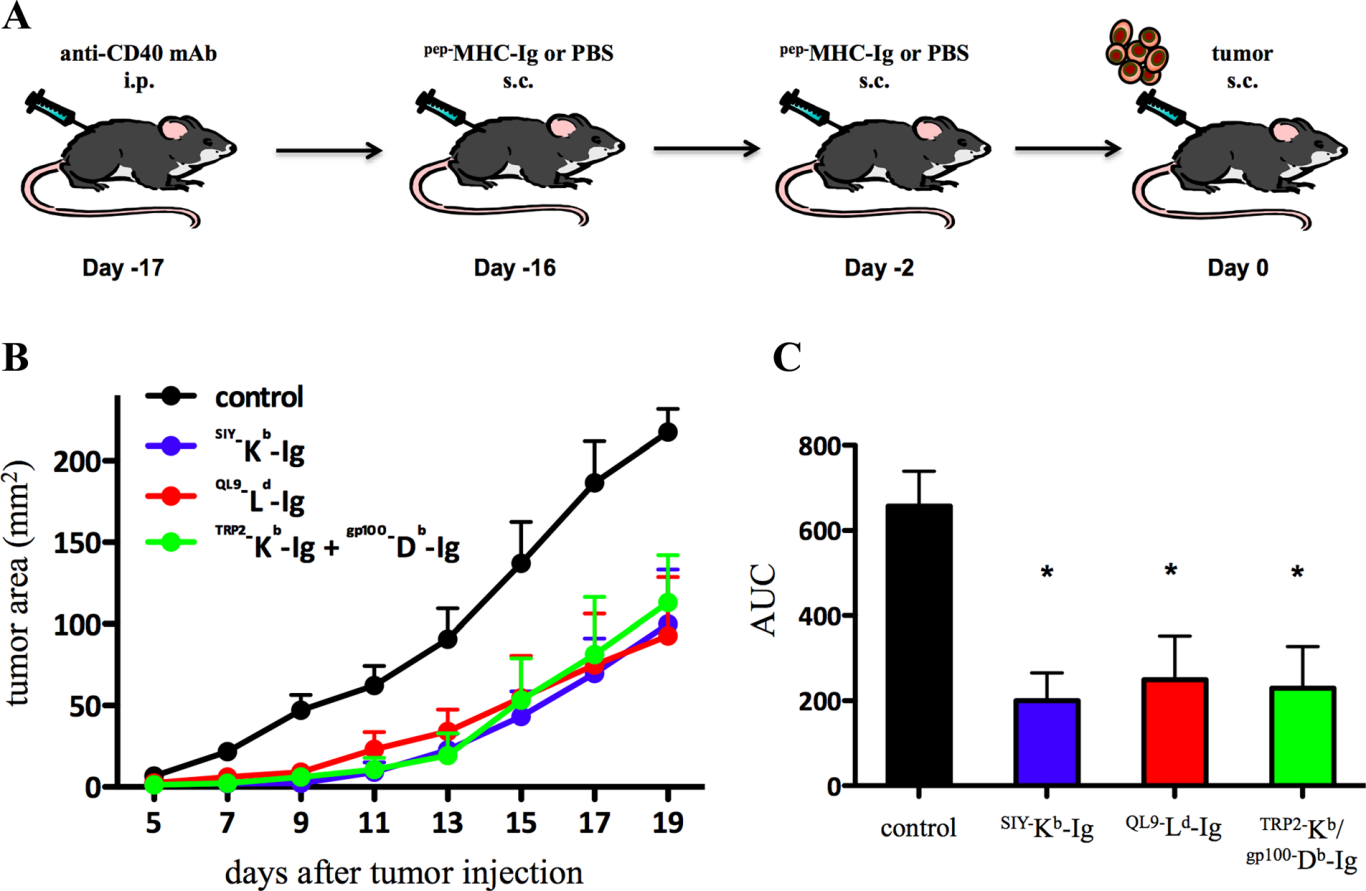
Inhibition of B16-SIY tumour growth in ^SIY−^K^b^-Ig immunized mice. (A and B) On day −17 mice were injected intra peritoneal (i.p.) with anti-CD40 mAb. One day later mice were s.c. immunized with ^SIY−^K^b^-Ig (blue line), ^QL9−^L^d^-Ig (red line) or a combination of ^TRP2−^K^b^-Ig and ^gp100−^D^b^-Ig (green line). On day −2 mice were boosted with additional ^pep−^MHC-Ig and subsequently injected with B16-SIY tumour cells on day 0. The control group was treated with anti-CD40 mAb and PBS injections (black line). Tumour size was measured as surface area (mm^2^) at indicated days. The area under curve (AUC) is displayed in (C). (C) Significance was calculated over the entire experiment by AUC for each mice (*P* < 0.05 by ANOVA with Tukey's post-test) * indicates significant difference of each treatment group (blue, red and green bar) from control group (black bar).

The effects of ^pep−^MHC-Ig immunization were also analyzed in a melanoma tumour prevention model. Wild type B6 mice were pretreated with anti-CD40 (day −17) and one day later (day −16) immunized with 250 µg/mouse ^SIY−^K^b^-Ig, ^QL9−^L^d^-Ig or a combination of ^TRP2−^K^b^-Ig and ^gp100−^D^b^-Ig dimer. On day −2 animals received an additional booster immunization with 50 µg/mouse ^pep−^MHC-Ig and B16-SIY tumour cells were administered on day 0 s.c. (Fig. 6A). Mice that were immunized with different ^pep−^MHC-Ig showed a delayed tumour growth with a statistically significant difference already seen at day 7. By day 19 mice immunized with ^QL9−^L^d^-Ig had the smallest tumour burden, with an average tumour size of 92.76 mm^2^ (±88 mm^2^), followed by 99.82 mm^2^ (±82 mm^2^) for mice treated with ^SIY−^K^b^-Ig and 113.08 mm^2^ (±71 mm^2^) for ^TRP2−^K^b^-Ig and ^gp100−^D^b^-Ig treated mice (Fig. 6B). Control animals showed the highest tumour burden of 217.51 mm^2^ (±32 mm^2^). Total tumour growth, seen as area under the curve (AUC), was significantly lower** (*P* < 0.05) for ^SIY−^K^b^-Ig (200.25 ± 161 mm^2^), ^QL9−^L^d^-Ig (249.58 ± 252 mm^2^) and ^TRP2−^K^b^/^gp100−^D^b^-Ig (229.42 ± 242 mm^2^) immunized mice than animals from the control group (657.76 ± 182 mm^2^) (Fig. 6C). Thus, ^SIY−^K^b^-Ig, ^QL9−^L^d^-Ig and ^TRP2−^K^b^/^gp100−^D^b^-Ig immunization induces a protective endogenous T cell response that has a significant anti-tumour activity in a B16-SIY melanoma model.

## Discussion

Over the last two decades induction of T cell immunity utilizing soluble ^pep−^MHC-Ig *in vivo* has been a challenging approach resulting in different outcomes depending on the experimental set up and treatment protocol [15,17,20,21].

Here we demonstrate that ^pep−^MHC-Ig can be used to induce a functional antigen-specific T cell response. In a 2C T cell based adoptive transfer model utilizing cognate high affinity ^SIY−^K^b^-Ig dimer molecules we have been able to induce a functional immune response (Fig. 1B, left half). A single application of ^SIY−^K^b^-Ig dimer led to a significant dose dependent increase in 2C T cell numbers with a peak at day 4–5.

While previous studies by Cai et al. and others on ^pep−^MHC-Ig molecules showed conflicting results regarding the requirement of co-stimulation for the expansion of 2C T cells [18,28–31] it is broadly accepted that the induction of a functional, long lasting memory CD8^+^ T cell response is dependent on co-stimulation and CD4^+^ T cell help [32]. It has been shown that CD8^+^ T cell responses are in part regulated by CD4 mediated CD40/CD40L signalling on DC [2–4] and can be bypassed by administration of anti-CD40 mAb [33]. Consequently, pre-treatment of adoptively transferred mice with anti-CD40 mAb one day before immunization (Fig. 1A) enhanced ^SIY−^K^b^-Ig dimer induced 2C T cell proliferation up to eightfold (Fig. 1B, right half). Moreover, these cells were highly cytotoxic and displayed an activated phenotype (Fig. 2).

We further investigated the mechanism of the soluble dimer immunization induced T cell response and took advantage of the fact that the clonotypic TCR of 2C T cells can recognize two different ^pep−^MHC class I dimer molecules; the syngeneic MHC K^b^ loaded with the heteroclytic peptide SIY (SIYRYYGL) and the allogeneic MHC L^d^ with QL9 (QLSPFPFDL) that cannot be cross presented in B6 mice [19]. Our data clearly demonstrate that immunization with ^QL9−^L^d^-Ig specifically induces the activation and expansion of adoptively transferred 2C T cells, which can only occur due to direct interaction of the dimer with the TCR on 2C T cells. While the induced T cell expansion in response to the ^QL9−^L^d^-Ig immunization was quite impressive it was not as strong as detected in ^SIY−^K^b^-Ig immunized mice (Fig. 3). One possible explanation is the higher affinity of the SIY peptide for 2C TCR's, rendering the high affinity ^SIY−^K^b^-Ig more immunogenic than the slightly lower affinity ^QL9−^L^d^-Ig.

Importantly, even 30 days after initial ^SIY−^K^b^-Ig immunization, a significant amount of 2C T cells could still be detected by both antibody staining and *in vivo* killing assay (Fig. 4A). Furthermore, a re-challenge of immunized mice 30 days after the initial immunization induced a much faster T cell proliferation that peaked at day 2, hence 3 days earlier than in the initial immunization and staining with anti-CD44 and anti-CD45 proofed a CD44^high^/CD45^low^ T cell memory phenotype (Fig. 4B). Together these data indicated that ^pep−^MHC-Ig immunization leads to the generation of a long lasting, functional memory T cell response.

These findings are in line with work from Carey et al. [17] as immunization with class I ^pep−^MHC-Ig and co-treatment with LPS induced a robust tumour- and virus-specific T cell response leading to reduced tumour growth and protection from lethal viral challenge. In contrast, more recently it has been found in clinical studies that vaccination with peptide and incomplete Freud's adjuvant (IFA) leads to accumulation of CD8^+^ T cells at the antigen rich vaccination site and subsequent induction of T cell dysfunction and deletion [26,27]. Thus, we see a unique advantage in class I ^pep−^MHC-Ig immunization over currently used approaches to generate a fully functional memory T cell response.

We could demonstrate that successful in vitro expansion of SIY-specific T cells from immunized mice was dependent on cognate ^pep−^MHC-Ig immunization (Fig. 5A and B). In addition, we showed in an *in vivo* killing assay that ^SIY−^K^b^-Ig immunized mice were able to recognize and kill SIY peptide pulsed target cells in an antigen-specific manner (Fig. 5C and D). Thus, together these data demonstrate that ^pep−^MHC-Ig immunization induces an endogenous, antigen-specific T cell response. Functionally this endogenous T cell response induced by ^pep−^MHC-Ig immunization has significant anti-tumour activity. Pre-treatment with anti-CD40 mAb and vaccination with ^SIY−^K^b^-Ig as well as with ^TRP2−^K^b^/^gp100−^D^b^-Ig induces an endogenous SIY or tumour-antigen (TRP2/gp100) specific T cell response that significantly inhibited tumour growth in a B16-SIY melanoma mouse model (Fig. 6). Furthermore, immunization with the allo-antigenic ^QL9−^L^d^-Ig resulted in a significant tumour inhibition demonstrating that the anti-tumour effects are rather due to direct TCR ^pep−^MHC-Ig interactions than to enhanced cross-presentation of the peptide on host MHC. Thus, we conclude our findings are in line with data from Carey et al. [17], while in contrast other studies by Cho et al. [34] using peptide immunization in conjunction with anti-CD40 mAb demonstrated only moderated anti-tumour effects. While our *in vivo* studies were of preventive nature currently ongoing experiments addressing the question if ^pep−^MHC-Ig dimer immunization induced T cell responses demonstrate the same efficiency on already established tumours and if anti-CD40 mAb can be efficiently substituted to avoid potential generation of auto-reactive T cell responses.

In summary, we have demonstrated that ^pep−^MHC-Ig immunization together with anti-CD40 treatment induces a robust antigen-specific, cytolytic T cell response including the formation of a long lasting memory T cell population. Mechanistically we have shown that this antigen-specific T cell induction is mediated through direct TCR ^pep−^MHC-Ig interaction. In addition, we have demonstrated that ^pep−^MHC-Ig immunization of naïve mice can induce a protective anti-tumour response from the endogenous T cell repertoire. These studies highlight the potential of ^pep−^MHC-Ig based approaches in the development of immunotherapy for cancer and infectious diseases.

## Material and Methods

### Mice and peptides

Eight week-old female C57BL/6 (H-2^b^; B6) mice were purchased from Jackson Laboratories (Bar Harbor, ME). 2C TCR Rag^−/−^ transgenic mice were maintained as heterozygotes by breeding on a C57/BL6 background in the Johns Hopkins animal facilities. Heterozygous 2C males were mated to wild type B6 females. Heterozygous 2C mice were identified by flow cytometry analysis, utilizing PBMC double stained with anti-2C TCR mAb (clone 1B2 FITC) and anti-CD8 (PECy5; BD Pharmingen, San Jose, CA, USA). Procedures involving animals and their care were in conformity with institutional guidelines that comply with national and international laws and policies. Peptides SIY (SIYRYYGL), SIIN (SIINFEKL), QL9 (QLSPFPFDL), TRP2 (SVYDFFVWL), gp100 (KVPRNQDWL), and mCMV (YPHFMPTNL) were purchased from Genscript (Piscataway, NJ).

### Dimer preparation

MHC-Ig dimers, K^b^-Ig, D^b^-Ig, and L^d^-Ig were loaded with peptide as described previously [24]. Briefly, dimer molecules were loaded with peptide by stripping at alkaline (pH 11.5) or mildly acidic (pH 6.5) conditions and then refolded in the presence of 40-fold excess peptide and twofold molar excess of human β_2_-microglobulin [35]. Unless otherwise indicated, ^SIY−^K^b^, ^SIIN−^K^b^, ^gp100−^D^b^-Ig, ^QL9−^L^d^, and ^mCMV−^L^d^ refer to MHC-Ig dimer reagent loaded with the indicated peptide.

### 2C CD8^+^ isolation and adoptive transfer

CD8^+^ T cells were enriched from 2C splenocytes by negative selection using a CD8^+^ T cell isolation kit (Miltenyi Biotec, Auburn, CA) according to the manufacture's instruction. After the negative isolation the CD8^+^ T cells were stained with the anti-2C TCR-FITC mAb (clone 1B2), anti-CD3-PE (BD-Pharmingen) and anti-CD8-PECy5 (BD-Pharmingen) to evaluate the purity of the population. The isolated 2C CD8^+^ T cells (3 × 10^6^) were adoptively transferred by i.v. injection in naïve B6 mice.

### Immunization protocol

Two days after adoptive transfer of 2C CD8^+^ T cells, recipient B6 mice were injected i.p. with 10 µg/mouse of anti-CD40 mAb (clone 3/23; BioLegend, San Diego, CA) and a day later immunized s.c. with the indicated amounts of either ^SIY−^K^b^-Ig, ^SIIN−^K^b^-Ig, ^gp100−^D^b^-Ig, ^QL9−^L^d^-Ig, or ^mCMV−^L^d^-Ig dimer.

### Expansion, activation and memory marker expression analysis

The 2C CD8^+^ T cell *in vivo* expansion was analyzed according to the kinetic displayed in Figure 1. PBMC of ^pep−^MHC dimer immunized mice were double stained with anti-2C TCR-APC mAb (clone 1B2) and anti-mouse anti-CD8 FITC (BD/Pharmingen). On day 5 and 30 after dimer injection the PBMC were analyzed for activation marker expression using the following anti-mouse mAb: CD25, CD44, CD62L, CD69, and CD122. The analysis was performed on 1B2^+^/CD8^+^ gated cells. Isotype controls matching to each mAb were used as negative control. All mAb used for phenotype analysis were PE labelled (BD-Pharmingen). Samples were read using the FACSCalibur (BD) and data analyzed by FACSExpress 3 (De Novo Software, Ontario, Canada).

### In vitro killing assay

Cytotoxic activity of CD8^+^ T cells was measured by 5 h ^51^Cr release assay using triplicate cultures in V-bottom plates. 0.2 × 10^6^/plate peptide pulsed (SIY, SIIN) T2-K^b^ target cells were loaded with 200 µCi ^51^Cr at 37°C for 1 h. E:T ratios were 1:1, 10:1 and 25:1 on 2000 target cells/well. To allow proper cell contact plates were spun down (300*g*, 5 min) right before incubation. Triplicate wells were averaged and percentage specific cytotoxicity was calculated as [(cpm sample − cpm spontaneous release) 100×/(cpm maximum release − cpm spontaneous release)]. For spontaneous release T2-K^b^ target cells were plated without CD8^+^ T cells in complete RPMI media. For maximum release target cells were plated with 0.15% Triton-X-100 (Sigma, St. Louis, MO).

### *In vivo* killing assay

Target cells for the *in vivo* cytotoxic assay were obtained from splenocytes of naïve B6 mice, cleaned from erythrocytes by osmotic lysis, washed and split into two populations. One population was pulsed with 1 µM SIY peptide, incubated at 37°C for 45 min, and labeled with a high concentration of CFSE (2.5 µM) (^SIY−^CFSE^high^ cells). The second control target population was pulsed with 1 µM SIIN peptide and was labeled with a low concentration of CFSE (0.25 µM) (^SIIN−^CFSE^low^ cells) (Invitrogen, Eugene, OR). The two populations were mixed together at 1:1 ratio and i.v. injected in ^pep−^MHC dimer immunized B6 mice (5 × 10^6^/population/mouse). After 18 h the mice were sacrificed and spleen, ^pep−^MHC dimer immunization site draining lymph node, and other lymph nodes were harvested. The cell suspensions obtained from each organ were analyzed by FACS for presence of two differentially CFSE labeled target populations. The recovery and percent killing of the various CFSE-labeled, peptide-pulsed target cells were calculated as follows: % of *in vivo* killing = 100 − ([(% specific peptide pulsed cells in immunized B6/% unspecific peptide pulsed cells in immunized B6)/(% specific peptide pulsed in naïve B6/% unspecific peptide pulsed cells in naïve B6)] × 100).

### *In vivo* tumour inhibition experiment

On day −17 B6 mice were injected intra peritoneal (i.p.) with 10 µg/mouse anti-CD40 mAb (clone 3/23; BioLegend). One day later (day −16) mice were subcutaneously (s.c.) immunized with 250 µg/mouse ^SIY−^K^b^-Ig, ^QL9−^L^d^-Ig, or a combination of ^TRP2−^K^b^-Ig and ^gp100−^D^b^-Ig. Fourteen days later (day −2) these mice received a boost s.c. injection of an additional 50 µg/mouse ^pep−^MHC-Ig dimer. All mice from control groups received s.c. PBS injections. Finally, on day 0 all mice were injected s.c. with 1 × 10^6^ B16-SIY tumour cells (generously provided by T. F. Gajewski and C. Blank). Tumour growth was monitored every other day utilizing a digital caliper. Mice were euthanized when tumour sizes reached >200 mm^2^. Tumour growth for each mouse was summarized as area under curve (AUC) and statistical analysis was performed in GrapPad Prism5.
